# Increasing clinical, community, and patient-centered health research for preventing and managing multimorbidity

**DOI:** 10.15256/joc.2013.3.26

**Published:** 2013-12-24

**Authors:** Jose M. Valderas

**Affiliations:** ^1^NIHR PenCLAHRC, Institute for Health Services Research, Medical School, University of Exeter, Exeter, UK; Health Services and Policy Research Group, Department of Primary Care Health Sciences, University of Oxford, Oxford, UK

**Keywords:** multimorbidity, multiple chronic conditions, comorbidity, health research, healthcare services

## Abstract

The report “Multiple Chronic Conditions: A Strategic Framework,” which was developed by the U.S. Department of Health & Human Services (HSS), has identified as one of the key goals for improving health and the provision of healthcare for people with multiple chronic conditions “to increase clinical, community and patient-centered research.” In their linked commentary of this special journal issue, Parekh and Goodman identify and consider the potential impact of a number of related research initiatives supported by the National Institutes of Health and the Agency for Health Research and Quality, particularly focusing on two very specific areas: behavioral medicine and secondary analyses of available datasets. In this paper, I comment on both documents and discuss the opportunities offered by the current approaches and highlight related research needs; in particular, the need for an improved and expanded conceptual model of healthcare for people with multimorbidity, and the need for further exploration of the use of multimorbidity-relevant outcomes as part of usual clinical practice.

Journal of Comorbidity 2013;3:41–44

## Introduction

The report “Multiple Chronic Conditions: A Strategic Framework,” which was developed by the U.S. Department of Health & Human Services (HSS), has identified as one of the key goals for improving health and the provision of healthcare for people with multiple chronic conditions (MCCs) “to increase clinical, community and patient-centered research” [1]. In a linked commentary in this special issue, Parekh and Goodman have considered the potential impact of a number of related research initiatives supported by the National Institutes of Health (NIH) and the Agency for Health Research and Quality (AHRQ), particularly focusing on two very specific areas: behavioral medicine and secondary analyses of available datasets [2]. In this paper, I comment on both documents and discuss the opportunities offered by the current approaches and highlight related research needs.

## The report

The U.S. Department of Health & Human Services (HHS) report “Multiple Chronic Conditions: A Strategic Framework,” identifies research as one of the four key goals for improving health and healthcare for people with multimorbidity. In particular, the report provides recommendations for facilitating “research to fill knowledge gaps about, and interventions and systems to benefit individuals with multiple chronic conditions.” Other goals are related to changes in the organization and delivery of healthcare, maximizing benefit from available systems (with a particular focus on self-management) and furnishing health and social care professionals working with patients with MCCs with specific resources [1]. The most ambitious objective associated with this research goal is “to increase clinical, community and patient-centered research.” This becomes clear when considering the narrower focus of the other three objectives: (1) increasing the validity of randomized controlled trials, (2) understanding the epidemiology of MCCs, and (3) addressing disparities [1].

The report acknowledges that there is insufficient evidence for both the impact of MCCs on health status and the treatment of MCCs. Indeed, a recent systematic review and meta-analysis of interventions for people with multimorbidity identified ten studies involving multifaceted complex interventions [3]. The results were mixed and non-conclusive, but suggested that interventions focusing on particular risk factors or functional difficulties are likely to be the most effective. The HHS report makes an explicit call for research on prevention, management, and treatment of MCCs. To address this, the report calls for an increased focus on research activity, on clinical-, self-care-, and community-based approaches for health promotion, disease prevention, and healthcare management of individuals with MCCs, as well as on the systems to best support and sustain this programing. The existence of current disincentives for providers to adequately address the needs of individuals with MCCs is also recognized in the report, and so is the need for identifying which outcomes are of highest importance to individuals with MCCs in order to enable clinicians to orient care towards them, calling for the development of innovative and reliable methods for researchers to improve measurement of patient-centered outcomes of treatments and other interventions for individuals with MCCs. Finally, a third relevant area is identified, namely the characterization of patient trajectories in relation to changes in health status, functional status, and health services research.

Although there is no attempt in the report to clarify the issue, the call for research on clinical, community, and patient-centered aspects of health and healthcare for patients with MCCs does not identify distinct areas of work, but rather overlapping approaches to the relevant aspects. In fact, patient centeredness is usually defined as the provision of care “that is respectful of and responsive to individual patient preferences, needs, and values, and ensuring that patient values guide all clinical decisions” (Institute of Medicine); community care is about the provision of clinical care in the community, with the aim of having an impact on the closest environments of each patient, and the definition of the ‘patient-centered medical home’ makes explicit references to the inclusion of community services and interventions [4], to consider but a few instances that demonstrate the deep interrelationship between these approaches.

## Research initiatives

In their linked commentary, Parekh and Goodman identify a number of related research initiatives supported by the NIH and the AHRQ [2]. Specifically, they identify four funding opportunities announced by the NIH in the period 2010–2013 aimed at fostering extramural research in two very specific areas: behavioral medicine and secondary analyses of available datasets. Behavioral approaches are related both to behavioral treatments and more generally to research methods and measures for conceptualizing, triaging, and assessing health behaviors (e.g. concordance and adherence to treatment, mental health problems, diet and exercise, and substance use/abuse disorders) and behavioral interventions. Secondary data analyses focus mostly on specific combinations of MCCs or medications for assessing costs, differences in effectiveness and safety of different treatment regimens, risks for specific beneficial and/or adverse health outcomes, interactions among medications, disease processes, and health outcomes, and generally methodological issues relevant to the analyses of the health impact or treatment of MCCs. The AHRQ, on the other hand, has led to the creation of the “MCC Research Network,” with a focus on comparative effectiveness, quality improvement, and patient-centered outcomes research. Both institutions and the Patient-Centered Outcomes Research Institute convened a meeting focusing on the health of people with MCCs in which contextual factors and relevant research methods were explored, and a research agenda was developed.

Parekh and Goodman conclude that the Strategic Framework provides a useful roadmap to foster coordination between HHS operating divisions and enhance collaboration with external stakeholders in MCC research [2]. They consider that HHS agencies and programs have productively used the framework to guide new research initiatives which, among others, have supported studies to improve the quality and effectiveness of medical management of persons with MCCs, while at the same time highlighting additional priority areas, notably the characterization of research opportunities of huge potential (such as interventions aimed at patients with combinations of prevalent high-impact concordant conditions), the availability of local level data, or the tailoring of data for specific use, such as the development of comorbidity-sensitive clinical practice guidelines.

My colleagues and I agree with Parekh and Goodman that a common framework is of utmost importance for moving this field decisively forward [5]. The framework stops short of presenting a conceptual model that could guide the effort. This is also evidenced, as mentioned before, by the lack of clarity as to whether the call for research refers to distinct areas or (more likely) to overlapping approaches. A conceptual model has been developed as a result of the associated efforts [6], but it would be key for ensuring progress that this conceptual model is expanded in its scope, ambition, and relevance for healthcare systems worldwide (the current model has been specifically developed for developing and applying classification schemes for chronic conditions to data elements for studying and monitoring health conditions in the USA). The framework proposed in the report should also be reworked in light of the improved conceptual model. This would ensure a more consistent and structured approach to the identification of the research needs in this area.

## Outcome priorities

Perhaps one of the more interesting issues in the report is the recognition of the need for identifying which outcomes are of highest importance to individuals with MCCs. Although a body of qualitative work is building up aiming precisely at identifying the problems that people with MCCs face (which is starting to be summarized [7]), the issue of what the outcomes of interest are has not yet been sufficiently explored. This issue is not at all trivial. Patients may be potentially interested in process-oriented intermediate outcomes, such as increasing their ability to manage and cope with their conditions and their potentially competing demands, as well as their proper health outcomes such as symptoms, functioning, perceived health or health-related quality of life [8]. At the same time, the question seems to imply that there are more-or-less standard outcomes that can be used for evaluating the results for all patients with MCCs. The likely realization that outcomes may vary from patient to patient and the huge diversity of problems posed by different combinations of conditions and different individual preferences for outcomes has the potential for suggesting that a reorientation of the question is required. There is therefore the potential for processes to be developed that will in themselves allow the tailoring of outcomes for the desired processes of care [9]. Such processes will most likely need to be centered around the figure of the physician responsible for providing the bulk of care and/or for coordinating healthcare across the different conditions – a role most frequently played in the various systems by primary care physicians (general practitioners and family physicians in most countries, along with internists and pediatricians in the USA).

The promising potential for behavioral approaches to improve healthcare and outcomes in people with MCCs is recognized. Smoking and obesity have a shared etiological role in many conditions that may develop in the same individual, and they are particularly sensitive to such approaches along with broader public health interventions.

Parekh and Goodman very appropriately identify secondary data analyses as a priority. The amount of information on the epidemiology and health services and broader social implications of the simultaneous presence of MCCs that have already been collected as part of research studies, routine health surveys, and routinely collected data is huge. Proper analysis of these vast resources is a most efficient approach to advancing our knowledge in this area and any researcher with an interest in MCCs should seriously consider focusing on them as a first step [10, 11]. Funding sources should also stimulate this type of research, recognizing their prominent role, not least also for their potential for the generation of hypothesis in relation to best models of management.

## Conclusion

The initiatives described in the linked commentary demonstrate the commitment of the research community towards improving the evidence base for the management of people with MCCs. Although there has been progress on a number of issues, the astonishing truth is that still know very little is known about best practice for these patients, although they represent the group with highest impact on the health service and also one with worst outcomes. Priority areas in this respect would be the development of an improved and expanded conceptual model for the delivery of healthcare to people with multimorbidity, and the characterization of multimorbidity-relevant outcomes that could be used in clinical practice. Advancing this area of research is obviously a priority both in terms of costs and health impact, and will pave the ground for new models of care that truly link the most advanced and technologically sophisticated medical care with approaches to the delivery of healthcare that are person-centred and individually sensitive.

## References

[1] U.S. Department of Health & Human Services (2010). Multiple chronic conditions: a strategic framework. Optimum health and quality of life for individuals with multiple chronic conditions.

[2] Parekh AK, Goodman RA (2013). The HHS strategic framework on multiple chronic conditions: genesis and focus on research. J Comorbidity.

[3] Smith SM, Soubhi H, Fortin M, Hudon C, O’Dowd T (2012). Managing patients with multimorbidity: systematic review of interventions in primary care and community settings. BMJ.

[4] Collaborative patient-centered primary care (2007). Joint principles of the patient centered medical home.

[5] Valderas JM, Starfield B, Sibbald B, Salisbury C, Roland M (2009). Defining comorbidity: implications for the understanding of health and health services. Ann Fam Med.

[6] Goodman RA, Posner SF, Huang ES, Parekh AK, Koh HK (2013). Defining and measuring chronic conditions: imperatives for research, policy, programs, and practice. Prev Chronic Dis.

[7] Goncalves DC, Valderas JM, Ricci I, Rogers A (2012). The management of multimorbidity in clinical practice: patients and health professionals’ perspectives: a systematic review of qualitative studies. PROSPERO.

[8] Valderas JM, Alonso J (2008). Patient reported outcome measures: a model-based classification system for research and clinical practice. Qual Life Res.

[9] Valderas JM (2013). Getting impatient about person centred health care. Eur J Gen Pract.

[10] Barnett K, Mercer SW, Norbury M, Watt G, Wyke S, Guthrie B (2012). Epidemiology of multimorbidity and implications for health care, research, and medical education: a cross-sectional study. Lancet.

[11] Salisbury C, Johnson L, Purdy S, Valderas JM, Montgomery AA (2011). Epidemiology and impact of multimorbidity in primary care: a retrospective cohort study. Br J Gen Pract.

